# Confirming content validity of The Insomnia Daytime Symptoms and Impacts Questionnaire (IDSIQ) among adults with insomnia in four European countries

**DOI:** 10.1186/s41687-025-00972-4

**Published:** 2025-12-29

**Authors:** Andrea Phillips-Beyer, Ariane K. Kawata, Leah Kleinman, Antonio Olivieri

**Affiliations:** 1 Innovus Consulting Ltd, Wingrave, UK; 2 Evidera, Bethesda, MD USA; 3 Evidera, Seattle, WA USA; 4 Idorsia Pharmaceuticals Ltd, Hegenheimermattweg 91, Allschwil, 4123 Switzerland

## Abstract

**Background:**

The Insomnia Daytime Symptoms and Impacts Questionnaire (IDSIQ) v2.0 is a 14-item patient-reported outcome measure for assessing the daytime symptoms and impacts of insomnia. Content validity and measurement properties of the IDSIQ have been established among English-speakers. This study assessed content validity of translations of the IDSIQ, generated for use in clinical trials in non-English-speaking countries.

**Methods:**

This was a cross-sectional, non-interventional, qualitative study in adults with insomnia from Denmark, Germany, Poland, and Spain. During one-on-one telephone interviews in their local language, participants answered the IDSIQ questions and described their overall impressions of the measure’s clarity, length, recall period, and comprehensiveness, as well as the overall relevance of the IDSIQ items to their experience of daytime symptoms and impacts of insomnia. They also explained their interpretation of each IDSIQ item (i.e., what they were thinking when they answered the question) and provided feedback on item clarity and the response options. Interview transcripts were translated into English and analyzed using ATLAS.ti.

**Results:**

Twenty adults with insomnia were interviewed (five per study country, average age 46.5 years). Most participants were female (75%) and were currently receiving treatment for insomnia (70%). All participants reported that the IDSIQ instructions were clear; almost all participants reported that they understood the individual IDSIQ items (95%) and that the number of items was appropriate (95%). While 60% of participants considered “today” an appropriate recall period, 35% suggested that a longer timeframe could also be useful. Most participants reported that the IDSIQ was comprehensive in its coverage of insomnia-related experiences (80%) and that the IDSIQ items collectively were relevant to their experiences of insomnia (80%). For each item, ≥ 85% of participants felt that the wording was clear and ≥ 75% of participants would not make any changes to the response options.

**Conclusions:**

Overall, participants in European countries representing differing languages and cultures considered the IDSIQ clear, comprehensive, and relevant to their experience of daytime symptoms and impacts of insomnia. Moreover, participant feedback indicated understanding of individual items as intended. These findings support content validity of the IDSIQ after its translation into different European languages, as well as its use in future studies.

**Supplementary Information:**

The online version contains supplementary material available at 10.1186/s41687-025-00972-4.

## Abstract (plain language summary)

The “IDSIQ” is a questionnaire used in research to assess how insomnia (difficulty falling or staying asleep) affects people’s ability to function during the day. The IDSIQ was initially developed in English and then translated into Danish, German, Polish, and Spanish. The aim of this study was to find out whether patients with insomnia from Denmark, Germany, Poland, and Spain understood the IDSIQ in their mother tongue. The study also aimed to assess whether translations of the IDSIQ adequately capture patient experiences of how insomnia affects daytime functioning. We interviewed 20 adults with insomnia (five in each country) and asked them about various aspects of the IDSIQ, including whether it was clear and relevant to their experiences of how insomnia affects their lives. All the participants reported that the IDSIQ instructions were clear, and nearly all reported that they understood the IDSIQ questions. Twelve participants (60%) considered “today” an appropriate timeframe for assessing the daytime impact of insomnia. Sixteen participants (80%) reported that the IDSIQ items were relevant and covered how insomnia affects their daytime functioning. For each IDSIQ question, most participants reported that they fully understood the question. Overall, the patients we interviewed considered the IDSIQ to be clear, comprehensive, and relevant to their experiences of insomnia. These findings support use of the IDSIQ after translation into other European languages.

**Supplementary Information:**

The online version contains supplementary material available at 10.1186/s41687-025-00972-4.

## Introduction

The Insomnia Daytime Symptoms and Impacts Questionnaire (IDSIQ) v2.0 is a patient-reported outcome (PRO) measure that assesses the daytime symptoms and impacts of insomnia. It comprises 14 items grouped into three domains: Alert/Cognition (six items), Mood (four items), and Sleepiness (four items). Each item is scored on a 0–10 numeric rating scale (NRS) [4]. For nine items, a higher raw score indicates greater impairment, while five items are reverse-coded prior to scoring because a higher raw score indicates less impairment. The questionnaire is designed to be completed every evening with a recall period of “today”.

The IDSIQ was developed in US English, and its measurement properties were previously established using data from an open-label insomnia trial conducted in the US and Germany [4]. In this trial, patients received zolpidem 5 or 10 mg daily for two weeks and completed daily morning and evening electronic Diaries. The IDSIQ was part of the evening diary. Following the study, a subset of patients participated in exit interviews to elicit concepts defining their insomnia experience and to evaluate their understanding of the IDSIQ. The IDSIQ was translated into multiple languages prior to its subsequent inclusion as an outcome measure in two phase 3 insomnia trials of the orexin receptor antagonist daridorexant (NCT03545191 and NCT03575104) [2, 5]. To ensure harmonization and cultural equivalence across countries and languages, the different translations were linguistically validated using standard PRO translation guidelines, as recommended by ISPOR [8]. However, content validity of the IDSIQ based on patient input has only been established in English.

This study aimed to assess content validity of IDSIQ translations used in several key European countries represented in phase 3 insomnia trials of daridorexant conducted in adults with insomnia.

## Methods

### Study design

This was a cross-sectional, non-interventional, qualitative interview study and not associated with a clinical trial. Participants were recruited by clinical sites in four European countries: Denmark, Germany, Poland, and Spain. All study materials were approved by an ethics committee in each country prior to study initiation. Each interview participant provided written informed consent.

### Participants

Clinical sites screened and recruited adults (≥ 18 years) diagnosed with insomnia at least 6 months before screening (based on medical records or clinical databases) who were able to speak, read, and understand the official language (Danish, German, Polish, or Spanish), as applicable, based on the country where they were recruited. Potential participants were excluded if they had any medical or psychiatric condition that might interfere with study participation.

### Study procedures

A third-party vendor (FACITtrans) interviewed participants individually by telephone in their local language. Participants were provided with a paper copy of the IDSIQ to review prior to the interview. The interviews lasted up to 45 min, and participants were remunerated for their time.

Interviews were conducted using a semi-structured interview guide. Participants were first asked about their experiences and understanding of insomnia (results not reported). The main part of the interview focused on cognitive debriefing of the IDSIQ. Participants answered the IDSIQ questions (without being informed that the IDSIQ is typically completed daily as part of a clinical trial). They were then asked about their impressions of the measure’s clarity (overall and for each item), ease of completion, length, recall period, and comprehensiveness, as well as the overall relevance of the IDSIQ items. Participants were also asked to explain how they interpreted each IDSIQ item. They were further asked whether they would suggest any changes to the individual items or overall questionnaire. After the interview, participants completed a questionnaire covering socio-demographics and current severity and treatment of insomnia.

### Analyses

Audio recordings of the interviews were transcribed and translated into English. Content analysis of the English transcripts was conducted in ATLAS.ti (ATLAS.ti GmbH, Berlin, Germany) [3] using a coding dictionary developed based on themes and concepts that emerged during the interviews.

Descriptive statistics were computed using SAS^®^ version 9.4 or later (SAS Institute, Cary, NC, USA).

## Results

### Participant characteristics

Twenty adults with insomnia (five per participating country) were interviewed. The average age of participants was 46.5 years (range 25–94) (Table 1) and was slightly higher in Denmark than in other countries (58.6 years vs. 40.4–44.8 years). Most participants were female (*n* = 15, 75%) and well-educated (college/university or postgraduate degree: *n* = 15, 75%). Eleven participants (55%) were employed full-time.

**Table 1:** Demographic and clinical characteristics of the interview participants

	Total Sample *N* = 20	Denmark *N* = 5	Germany *N* = 5	Poland *N* = 5	Spain *N* = 5
**Age (years)**					
Mean (standard deviation)	46.5 (15.3)	58.6 (21.5)	44.8 (9.0)	40.4 (13.3)	42.0 (11.5)
Median (range)	45 (25–94)	50 (41–94)	47 (33–53)	36 (33–64)	45 (25–55)
**Gender**, **n (%)**					
Male	5 (25)	0	2 (40)	2 (40)	1 (20)
Female	15 (75)	5 (100)	3 (60)	3 (60)	4 (80)
**Highest level of education**, **n (%)**					
Elementary/primary school	1 (5)	1 (20)	0	0	0
Secondary/high school	1 (5)	0	1 (20)	0	0
Technical or vocational degree	3 (15)	1 (20)	0	1 (20)	1 (20)
College/university degree	8 (40)	2 (40)	1 (20)	1 (20)	4 (80)
Postgraduate degree	7 (35)	1 (20)	3 (60)	3 (60)	0
**Employment status**,** n (%)**					
Full-time work	11 (55)	2 (40)	3 (60)	2 (40)	4 (80)
Part-time work	4 (20)	2 (40)	2 (40)	0	0
Other	5 (25)	1 (20)	0	3 (60)	1 (20)
**Self-rated general health in the past month**,** n (%)**					
Very good	1 (5)	1 (20)	0	0	0
Good	12 (60)	1 (20)	3 (60)	4 (80)	4 (80)
Fair	5 (25)	1 (20)	2 (40)	1 (20)	1 (20)
Poor	2 (10)	2 (40)	0	0	0
**Self-rated current severity of insomnia**,** n (%)**					
Mild	4 (20)	2 (40)	1 (20)	1 (20)	0
Moderate	12 (60)	0	3 (60)	4 (80)	5 (100)
Severe	4 (20)	3 (60)	1 (20)	0	0
**Currently receiving treatment for insomnia**,** n (%)**					
No	5 (25)	0	3 (60)	1 (20)	1 (20)
Yes	14 (70)	4 (80)	2 (40)	4 (80)	4 (80)
Missing	1 (5)	1 (20)	0	0	0
**Current treatments**,** n (%)**^**1**^					
Cognitive behavioral therapy	7 (35)	1 (20)	2 (40)	1 (20)	3 (60)
Stimulus control therapy	1 (5)	0	0	0	1 (20)
Relaxation techniques	5 (25)	0	2 (40)	1 (20)	2 (40)
Sleep restriction	2 (10)	0	0	0	2 (40)
Light therapy	1 (5)	1 (20)	0	0	0
Prescription medications	7 (35)	0	2 (40)	2 (40)	3 (60)
Zolpidem	3 (15)	0	2 (40)	0	1 (20)
Other	4 (20)	0	0	2 (40)	2 (40)
Over-the-counter sleep aid	2 (10)	0	1 (20)	1 (20)	0
Lifestyle and home remedies	4 (20)	1 (20)	2 (40)	1 (20)	0
Alternative therapies	12 (60)	5 (100)	2 (40)	2 (40)	3 (60)
Melatonin	6 (30)	1 (20)	1 (20)	1 (20)	3 (60)
Valerian	4 (20)	2 (40)	1 (20)	0	1 (20)
Yoga or tai chi	2 (10)	1 (20)	0	1 (20)	0
Meditation	5 (25)	3 (60)	1 (20)	1 (20)	0
Other	4 (20)	0	1 (20)	0	3 (60)

^1^ Not mutually exclusive

Across countries, approximately two-thirds of participants rated their general health as “good” or “very good” (*n* = 13, 65%). Five participants (25%) rated their general health as “fair”, and two (10%) rated it as “poor.” Across countries, 60% of participants rated their current insomnia severity as “moderate”, 20% as “mild”, and 20% as “severe”. The participants who rated their insomnia as “severe” were from Denmark (*n* = 3) and Germany (*n* = 1).

Most participants (*n* = 14, 70%) were currently receiving one or more treatments for insomnia. The most commonly reported insomnia treatments were alternative therapies (*n* = 12, 60%), cognitive behavioral therapy (*n* = 7, 35%), and prescription medications (*n* = 7, 35%). Alternative therapies included melatonin (*n* = 6, 30%), meditation (*n* = 5, 25%), and valerian (*n* = 4, 20%). The most frequent type of cognitive behavioral therapy was relaxation techniques (*n* = 5, 25%). Three of the seven participants who reported using prescription medications were taking zolpidem.

### IDSIQ scores

As part of the item debriefing, participants answered each IDSIQ item using the 0–10 response scale. Overall, participants provided a range of responses across items. The lowest median scores were for item 6 (“How irritable did you feel today?”, where a lower raw score indicates less impairment) (3 [range 0–10]) and item 10 (“How refreshed did you feel today?”, where a lower raw score indicates greater impairment) (3 [range 2–6]) (Table 2). The IDSIQ item with the highest median score was item 11 (“How mentally tired did you feel today?”, where a higher raw score indicates greater impairment) (6.50 [range 1–8]).

**Table 2:** IDSIQ item scores reported by interview participants (*N* = 20)

	Median	Range (min-max)
Item 1: How clear-headed did you feel today?	6.0	3–10
Item 2: How well were you able to concentrate today?	6.0	3–9
Item 3: How forgetful did you feel today?	4.0	0–7
Item 4: How worried did you feel today?	4.0	0–9
Item 5: How frustrated by your lack of sleep did you feel today?	5.5	0–9
Item 6: How irritable did you feel today?	3.0	0–10
Item 7: How stressed did you feel today?	5.0	0–9
Item 8: How energetic did you feel today?	5.0	1–9
Item 9: How much of an effort was it to perform daily activities (i.e., reading, cleaning, work, school) today?	5.0	1–9
Item 10: How refreshed did you feel today?^1^	3.0	2–6
Item 11: How mentally tired did you feel today?	6.5	1–8
Item 12: How physically tired did you feel today?	6.0	2–9
Item 13: How sleepy did you feel today?	6.0	3–9
Item 14: How awake did you feel today?^1^	5.0	3–8

IDSIQ © 2020, University of Pittsburgh. All rights reserved. IDSIQ-14 was created in 2020 by Idorsia Pharmaceuticals Ltd under license and distributed by Idorsia Pharmaceuticals Ltd under license. IDSIQ is further a registered trademark of Idorsia Pharmaceuticals Ltd

IDSIQ items are scored on a 0–10 numeric rating scale, where 0 = “not at all” and 10 = “very” or “a lot.” A higher raw score indicates greater impairment, with the exception of items 1, 2, 8, 10, and 14, where a higher raw score indicates less impairment

^1^ Response for this item missing from one participant

### Clarity and ease of completion

All participants reported that the IDSIQ instructions were clear, and nearly all (*n* = 19, 95%) were able to read and understand all the items (one Danish participant expressed that the concept of clear-headedness (item 1) was unclear; see Table 3 for a sample of participant quotes relating to different aspects of the cognitive debriefing). Nineteen participants confirmed that they could answer all the IDSIQ questions (one participant was not asked this question). In discussing the response options, one participant highlighted the need to be careful when answering the questions because a rating of 10 on the 0–10 scale represents the highest level of impairment for some items and no impairment for other items (Table 3).

**Table 3:** Example participant quotes on the IDSIQ

Aspect	Participant quote [unique participant ID, country]
**Overall clarity**	INTERVIEWER: And what did you think of the instructions in the questionnaire? “*Instructions? What do you mean by ‘instructions’?*” INTERVIEWER: Like, for example, “Please choose a number from a scale of zero to ten.” “ *Yeah*,* you can’t misunderstand that. That’s good.*” [300-005, Germany]
	“*…it was very clearly worded […] the […] questions*,* they are very understandable and simple.*” [400-001, Poland]
	“*…I understood everything.*” [400-003, Poland]
**Number of questions**	INTERVIEWER: And what did you think of the length? “*It was okay. But it should not be any longer.*” [300-002, Germany]
	“*I thought it was fine. Not too long or excessively short.*” [500-002, Spain]
**Recall period**	INTERVIEWER: And do you think “today” is an appropriate time period? “*Yes. […] It’s a snapshot then.*” [300-004, Germany]
	“*That could certainly be extended a bit*,* the survey period. ‘Today’ is of course only a momentary snapshot. I think it could definitely ask for a longer period of time.*” INTERVIEWER: What would you suggest? “*14 days.*” [300-005, Germany]
**Comprehensiveness**	INTERVIEWER: How much does this reflect your insomnia-related experiences? “*It reflects them well.*” [400-002, Poland]
	INTERVIEWER: How well does this questionnaire cover your experience of insomnia? Is there anything missing? “*No*,* I think it’s pretty good. It covers things that even I hadn’t considered.*” [500-003, Spain]
**Overall item relevance**	INTERVIEWER: And were the questions relevant to your experiences with insomnia? “*Yeah*,* sure. Totally.*” [300-003, Germany]
	“*I wouldn’t always associate the questions with insomnia. […] ‘Irritation’ in question 6 is only related to insomnia*,* I think*,* due to the general influences such as work*,* telephone*,* cell phone*,* emails*,* etc…. […] So this question is too… it doesn’t reflect the field of influencing factors.*” [300-005, Germany]
	“*I was more expecting questions maybe … strictly*,* how my insomnia manifests itself … and here there are questions only about what it causes during the day.*” [400-004, Poland]
**Suggested changes**	INTERVIEWER: Is there anything you would like to change regarding the response options? “*…You have to naturally be a little careful sometimes*,* as 10 is the most negative and other times 10 is the most positive. […] But when there aren’t a lot of questions you’re going to sit and read more thoroughly than if there were 30 questions […] So*,* maybe that’s not a problem.*” [100-002, Denmark]
	INTERVIEWER: And would you change anything in the questions or in the responses on this scale from 1 to 10? “*No*,* it’s adequate*,* everything is…*” [400-005, Poland]
	INTERVIEWER: Would you make any changes to the items or response options? “*No*,* it seemed quite appropriate*,* really. No*,* I think it’s good.*” [500-004, Spain]
	“*…being able to explain something further or asking an open question at the end. […] I think that would be interesting.*” [500-005, Spain]
**Feedback on individual items**	
Item 1 (clear-headed)	“*…‘clear-headed’ … it is general […] I wasn’t completely […] clear about what the definition was.*” [100-001, Denmark]
	“*What [do] you actually mean by ‘being clear-headed’? […] What [are] you actually trying to say? Foggy? […] [If] I was under the influence of medication. Then I could imagine that would actually affect me. Then I could imagine with such a medication*,* that I would not be clear-headed. But normally…? I may be tired*,* but I’m still clear-headed.*” [300-005, Germany]
Item 5 (frustrated)	“*…the word ‘frustrated’ is so incredibly broad*.” [300-002, Germany]

Each IDSIQ item was reported as being clear and understandable by at least 85% of participants. Only three reports indicated item ambiguity: two participants (one each from Denmark and Germany) for item 1 (“How clear-headed did you feel today?”) and one German participant for item 5 (“How frustrated by your lack of sleep did you feel today?”) (see Table 3 for quotes from these participants). Moreover, for each item, at least 75% of participants would not make any changes to the response options.

### Number of questions

Almost all participants (*n* = 19, 95%) reported that the IDSIQ has an appropriate number of items. In commenting on the length of the IDSIQ, one participant noted that the IDSIQ by itself may not be comprehensive, because it does not capture all concepts related to the experience of insomnia.

### Recall period

Over half of participants (*n* = 12, 60%) reported that “today” was an appropriate timeframe for the IDSIQ items. Longer timeframes of one to two weeks or more were proposed by seven participants (35%). All participants understood the concept of “today”.

### Comprehensiveness

Most participants (*n* = 16, 80%) reported that the IDSIQ was comprehensive in its coverage of their insomnia-related experiences. Individual participants (*n* = 1, 5% per suggestion) proposed including descriptions of specific sleep problems; including causal or differentiating factors related to insomnia; capturing factors such as nutrition, hydration status/fluid intake, and exercise/sports that can play a role in sleep; and asking about the type of insomnia experienced. Conversely, one participant suggested excluding non-sleep-related causes of daytime symptoms and impacts.

### Overall item relevance

Most participants (*n* = 16, 80%) found the IDSIQ items relevant to their experiences of insomnia. One participant (5%) reported that the items were “somewhat” relevant, noting that, for some items, such as irritability, the context could be difficult to parse out from the overall experience of the emotion in relation to insomnia (Table 3). No other participants provided similar feedback on overall item relevance.

## Discussion

In answering the IDSIQ items, participants provided a range of responses across items, reflecting adequate use of the full 0–10 NRS. Items were generally interpreted as intended, and the recall period was deemed appropriate by the majority of participants. Overall, the IDSIQ was concluded to be comprehensive in capturing daytime impacts and impairment due to insomnia.

Several participants suggested changes to capture additional aspects of their insomnia experience. Because we aimed to capture participants’ unfiltered views on the IDSIQ, no other sleep measures were discussed. It is therefore understandable that participants might expect the IDSIQ to fully capture their experiences with insomnia. In clinical trials, we would expect the IDSIQ to complement a sleep assessment using a daily sleep diary as part of a comprehensive insomnia measurement strategy. This was not discussed with participants. Moreover, the participants who proposed a longer recall period for the IDSIQ may not have been aware that it was developed for daily completion to capture fluctuations in daytime symptoms and impacts.

One participant identified reverse scoring of some items as a possible source of confusion. Inclusion of positive- and negative-worded items in PRO measures such as the IDSIQ is intended to reduce response bias and acquiescence. Van Sonderen et al. [6] cautioned that reverse wording of questionnaire items can cause data issues because of “respondent inattention and confusion” [6]. Various methods can be used to help visually distinguish negative and positive items [7]. In the IDSIQ, bold text and verbal descriptors at 0 and 10 on the NRS help guide respondents in scoring each item [4].

In evaluating interpretation of individual items across countries, it was noted that, compared to participants from other countries, participants from Germany tended to be more literal in their item interpretation and focused on items within the context of job performance and task completion. Some additional cultural nuances in item interpretation, not seen in other countries, were also observed among a few participants from Germany. One participant found it difficult to interpret item 1 (clear-headed), although they were unable to suggest how the wording might be improved. Another participant suggested that the meaning of clear-headedness could be clarified by providing examples in parentheses. In discussing item 5 (frustrated), the same two participants (both of whom were > 50 years) expressed that the word for frustrated should be changed. These and one additional participant had used the word “annoyed” when describing their interpretation of this item. In follow-up discussions after the interviews, German translation experts (FACITtrans; personal communication) advised that the word for “frustrated” used in the German version of the IDSIQ (“*frustriert*”) was not widely used in everyday speech thirty years ago. They recommended that no changes be made to this item, explaining that although older people may not be as familiar with this term, it is now in mainstream use and a good fit for the original English item. Based on findings from this study, it was concluded that the German translations of items 1 and 5 did not need to be changed.

The study has several limitations. Firstly, the study sample size was relatively small, with 5 participants each from 4 European countries. Although ISPOR recommendations and established qualitative research practices (e.g., COSMIN), suggest that as few as 4 or 5 participants may be adequate to confirm conceptual equivalence [8], additional research with a larger sample, more demographically diverse participants, and other European countries could confirm our findings and provide further insights. While the sample is small, this study yielded highly relevant insights into comprehension, interpretation, and cultural nuance, supporting the instrument’s content validity across 4 European populations and its clinical applicability in multinational insomnia studies.

Secondly, although insomnia is more common among women and older individuals, the cognitive debriefing sample in this study was predominantly female and highly educated, which may limit demographic representativeness. However, as the objective was to assess comprehension and conceptual equivalence rather than estimate symptom prevalence, the potential influence of these factors on the findings is likely minimal [8].

Lastly, as participants were not informed that the IDSIQ is intended to be used alongside a daily sleep diary and other sleep-related assessments in clinical trials, their judgments of item relevance and recall period may have differed from those in an actual trial setting; however, given the context-independent nature of the concepts assessed, we expect minimal impact on generalizability.

## Conclusion

Overall, participants considered the IDSIQ to be clear, comprehensive, and understandable, and consistently interpreted the items as intended. Moreover, although the IDSIQ included positively and negatively worded items, participants understood and used the corresponding response scales appropriately. Collectively, participants also provided answers that utilized each level of the NRS. Some participants provided constructive feedback, such as suggesting that additional items or descriptions/examples be included. Since the IDSIQ focuses only on daytime impacts, it was not designed to take the place of a daily sleep diary recording participant’s sleep patterns. In the intended context of use for the IDSIQ, the majority of their suggestions could be captured in a clinical study by administering the IDSIQ alongside a daily sleep diary or another insomnia instrument such as the Insomnia Severity Index [1] to comprehensively capture all concepts relevant to insomnia. Participants provided little or no critical feedback that indicated a lack of clarity or comprehension of the instrument in the languages tested. These findings support the view that IDSIQ translations in other European languages, developed using standard methods for translation and cultural adaptation [8], may have similar performance and be appropriate for use in future studies.

## Supplementary Information

Below is the link to the electronic supplementary material.

Supplementary Material 1

## Data Availability

The datasets used and/or analyzed during the current study are available from the corresponding author on reasonable request.

## Abbreviations

IDSIQ: Insomnia Daytime Symptoms and Impacts Questionnaire
NRS: Numeric rating scale
PRO: Patient-reported outcome
