# Supplementary material for: Confirming content validity of The Insomnia Daytime Symptoms and Impacts Questionnaire (IDSIQ) among adults with insomnia in four European countries
Source: J Patient Rep Outcomes. 2025 Dec 29;9:143. doi: 10.1186/s41687-025-00972-4 (PMC12748474; doi:10.1186/s41687-025-00972-4)
Supplement: Supplementary file 1 — Supplementary Material 1 [file 41687_2025_972_MOESM1_ESM.docx]

**Supplementary Material**

**Confirming content validity of the Insomnia Daytime Symptoms and Impacts Questionnaire (IDSIQ) among adults with insomnia in four European countries**

**Interview Guide**

***[Note: This interview guide is meant to help guide the discussion, but not to be used as a verbatim script; probes and questions may change slightly depending on individual feedback.]***

Introductory Script

Good (morning/afternoon/evening). My name is ______ and I am a [position] with FACIT, the company conducting this study. Thank you for agreeing to participate in this interview. Before we begin our discussion, I’d like to briefly tell you why we are conducting this interview and explain how our discussion today will work.

The overall purpose of the research study is to obtain feedback on a questionnaire designed for daytime symptoms and impacts of insomnia. During the course of the interview, I will also ask you to provide your thoughts on an insomnia questionnaire; the Insomnia Daytime Symptoms and Impacts Questionnaire (IDSIQ). I will ask you questions about the clarity, relevance of the questions, and the ease of selecting a response. ***[Confirm subject received the questionnaire].*** At the end of the interview, I will ask you to fill out a short questionnaire that will collect some basic information about you, such as your age, sex, and education.

Please know that there are no right or wrong answers in this interview; we are very interested in your personal experiences and opinions.

Do you have any questions so far? ***[If yes, answer questions.]***

I want to remind you that you are a volunteer in this study. If at any time you find a question difficult or challenging to respond to, you can ask for a short break or you can skip any question you do not want to answer, or stop the interview at any time. Also, I want to remind you that all your responses will be confidential.

I would like to ask for your permission to audio-record this interview. This recording will be kept confidential and your name will not be linked with the responses you provide. The recording will be used to ensure that we capture what you share, which will be important when we write a summary report of all data we have collected from people with insomnia. Do I have permission to audio-record this interview?

🞏 Yes

🞏 No ***[terminate the interview, audio-recording is an eligibility requirement.]***

Before we begin, let me suggest some things that will make our discussion more productive.

- Please speak up. We’re audio-recording the session because we do not want to miss any of your comments.
- We will be on a first-name basis, but in the study reports no names will be attached to any comments. Your name will be kept as confidential as possible, as discussed in the informed consent form.
- My role here is to ask questions and to listen. I won’t be actively participating in the conversation, only guiding it. I also may summarise information at times. I’ll ask questions about issues related to your experience and I’ll move the discussion from one question to the next to try to keep us on track so that we can finish in 45 minutes.
- I am not a medical doctor, so I am not qualified to give any medical advice. We encourage you to follow up with your regular doctor if you have any questions about your condition or treatment.
- You may choose not to participate in all or any part of the interview; if you withdraw, your current or future treatment will not be affected.
- You will receive 50 euros (or equivalent in local currency) after completion of the interview. I can give you more details about the compensation process at the end of the call.
- Please feel free to stop me at any time to ask me a question or to have me clarify something if you need to.

Do you have any questions before we begin? ***[If yes, answer questions.]***

**Begin Recorder:** This is study ***[insert project number here]*** and subject ID ***[insert ID number here]*** and today’s date is ***[insert date here].*** Do I have your permission to record this interview? I want to confirm that you completed the necessary consent form and that you have a copy of it for your records. Is that correct?

Ok, let’s get started.
